# Complete genome sequencing of SARS-CoV-2 strains: A pilot survey in Palestine reveals spike mutation H245N

**DOI:** 10.1186/s13104-021-05874-4

**Published:** 2021-12-23

**Authors:** Amer Al-Jawabreh, Suheir Ereqat, Kamal Dumaidi, Hanan Al-Jawabreh, Abedelmajeed Nasereddin

**Affiliations:** 1 Department of Medical Laboratory Sciences, Faculty of Allied Health Sciences, Arab American University, Jenin, Palestine; 2 Leishmaniases Research Unit, Jericho, Palestine; 3 Biochemistry and Molecular Biology Department, Faculty of Medicine, Al-Quds University, Abu Dis, East Jerusalem Palestine; 4 Al-Quds Nutrition and Health Research Institute Faculty of Medicine, Al-Quds University, Abu Dis, East Jerusalem Palestine

**Keywords:** SARS-CoV-2, Mutation, Palestine, COVID-19, Complete genome sequence, Spike-H245N

## Abstract

**Objectives:**

SARS-CoV-2, severe respiratory syndrome coronavirus-2, is an RNA virus that emerged from China sweeping the globe in the form of a pandemic that became an international public health concern. This pilot study aimed to describe the genetic variation and molecular epidemiology of SARS-CoV-2 in Palestine in fall 2020.

**Results:**

To achieve these aims, whole genome sequencing of SARS-CoV-2, phylogenetic analysis, haplotype networking and genetic diversity analysis were performed. These analyses revealed a unique spike mutation H245N that has never been reported before. The phylogenetic analysis depicted that three clusters existed in Palestinian SARS-CoV-2 genome sequences, in which cluster-I comprised the majority of clusters by 90%. Congruently, the haplotype network analysis depicted the same three clusters with a total of 39 haplotypes. The genetic diversity analysis showed that Cluster-I is highly diverse as confirmed by statistically significant mutation rate indices, Tajima’s D and Fu-Li’s-F tests (− 2.11 and 2.74, respectively), highest number of mutations (Eta = 120), highest number of haplotypes (h = 17), and highest average number of nucleotide differences between any two sequences (S = 118). The study confirmed the high genetic diversity among the Palestinian of SARS-CoV-2 which possessed high number of mutations including one which was reported for the first time.

**Supplementary Information:**

The online version contains supplementary material available at 10.1186/s13104-021-05874-4.

## Introduction

Severe acute respiratory syndrome coronavirus 2 (SARS-COV-2) is an enveloped, positive sense, single-stranded RNA virus, belonging to the family *Coronaviridae*, genus *Betacoronavirus.* SARS-CoV-2 was firstly identified and reported in Wuhan, the capital city of Hubei province in China, in December, 2019 and reported as the causative agents of the severe acute respiratory syndrome termed COVID-19 [1]. In Palestine including the West Bank, Gaza strip and East Jerusalem, the first cases were reported on March 4, 2020. The number has grown since then, reaching 452,274 laboratory-confirmed COVID-19 cases and 4651 deaths in October 2021 [2].

SARS-CoV-2 appears to have a relatively stable genome; however several lineages of the virus along with genetic diversities were reported that might be the consequences of uncontrolled spread of SARS-CoV-2 among humans within or beyond a territory [3]. A new variant of SARS-CoV-2 (lineage B.1.1.7) has recently been detected in England that has higher rate of transmissibility than the previous circulating strains, however, no evidence of increased clinical severity or vaccine escape capability reported about this variant [4]. Furthermore, another SARS-COV-2 variant (501Y.V2) was introduced by Tegally, et al. in South Africa that its transmissibility is 50% higher than previous variants [5]. Other recent variants of concern are from Brazil (B.1.1.28-484 K.V2 and B.1.1.248) [6]. The dynamic of SARS-COV-2 mutations highlight the importance of a global active-genomic surveillance of the virus and could lead to early identification and characterization of emerging variants prior any possible pandemics. Whole genome sequencing is currently used to study the genetic diversity of the emerging SARS-CoV-2 using various bioinformatics tools such as phylogenetic analysis, haplotype networking and genetic diversity indices [7]. The aims of this pilot study were to define the genetic structure of the SARS-COV-2 in nasopharyngeal samples collected from the SARS-COV-2 patients in the West Bank of Palestine through whole genome sequencing and characterize viral genomic variations, circulating mutations, and genetic relationships between local strains and a reference and selected global strains.

## Main text

### Materials and methods

#### Study population and sample collection

As part of a pilot descriptive cross-sectional study, nasopharyngeal swabs (Poctman, Guandong Poctman Life Technology Co., Ltd, China) were collected from 10 Palestinian patients after being officially confirmed as COVID-19 cases with varying degrees of classical symptoms including fever, cough, headache, and loss of smell. The median age of study patients was 30.5 years with four males included. The study samples were collected in November 2020 from seven Palestinian districts (Additional file 1). All Samples were transported at 4 °C in a sealed container for RNA extraction and processing.

#### RNA extraction and DNA Flex library preparation

Viral RNA was extracted using QIAamp® Viral RNA Extraction Kit (Qiagen Diagnostics GmbH, Germany) according to manufacturer's instructions. DNA library preparation and sequencing were carried out based on ARTIC v3 amplicon and IDT ARTIC nCoV-2019 respectively, as described before [8].

#### Viral whole genome assembly

FASTQ format files as output of sequencing were analyzed using galaxy program (Galaxy Version 0.7.17.1) (https://usegalaxy.eu/) [9]. SARS CoV-2 consensus sequences were obtained based on mapped reads via BWA-MEM—map medium and long reads (> 100 bp) against the Wuhan-Hu-1 SARS-CoV-2 reference isolate (hCoV-19/Wuhan/Hu-1/2019, Genbank accession number NC_045512.0, GISAID accession ID: EPI_ISL_402125) [10, 11].

### Population genetics analyses

The population genetics analyses included phylogenetic tree construction, haplotype network analysis, and genetic diversity analysis. The GISAID-deposited and GenBank-deposited study genomes along with randomly-retrieved genomes from the Genbank and GISAID were aligned using the MEGA version X [12]. Maximum likelihood (ML) trees of the complete genomes with 1000 iterations for bootstrapping were constructed using MEGA version X. The analysis was performed on ten Palestinian SARS-CoV-2 genomes, 32 retrieved genomes, Wuhan reference strain, and an out-group genome of the bat Coronavirus from China (Genbank: MN996532.2).

The PopArt 1.7 software was used to construct three types of haplotype network analyses; median-joining haplotype network, which is a common method to depict relationship between closely related DNA sequences of the same species or population [13, 14]. To estimate the relationship between haplotypes, the networks were analyzed based on country of origin of viral genomes using nexus input files produced by DnaSP version 6.12.03. As the number of characters of the genomes analyzed was approximately equal, default parameters of the software including epsilon value of zero were used [13].

Population nucleotide diversity indices included number of haplotypes (h), mean genetic diversity (Hd), nucleotide diversity per site (π), total number of mutations (Eta), and average number of nucleotide differences (k). Neutrality tests as estimators of mutation rate including Tajima’s D and Fu Li’s F tests and genetic differentiation parameters such as genetic differentiation index (Fst) and migration rate (Nm) were calculated using DnaSP ver. 6.12.03 [15].

#### Mutation analysis

Two online mutation analysis platforms were used; GISAID CoVsurver (https://www.gisaid.org/epiflu-applications/covsurver-mutations-app/) and Genome Detective virus tool version 1.132-Coronavirus typing tool (https://www.genomedetective.com/app/typingtool/cov/).

### Results

#### Mutation analysis

The first strain containing the unique spike mutation H245N (genotype: B 1.1.50) was isolated in Palestine in November 2020 (Genbank: MW419997, GISIAD: EPI_ISL_752605, hCoV-19/Palestine/AAS24/2020). This mutation was reported in the GISAID database (https://www.gisaid.org/) in December 2020 in 10 isolates from 3 countries: 8 Israeli (EPI_ISL_745067, EPI_ISL_804138, EPI_ISL_944468, EPI_ISL_944496, EPI_ISL_1073190, EPI_ISL_1073491, EPI_ISL_944398, EPI_ISL_944386), one Italian (EPI_ISL_837473), and one New Zealander strain (EPI_ISL_707806) [16]. By the end of March 2021, 50 H245N mutations were reported worldwide with 11 in USA (9 in Florida, one in California, and one in Massachusetts). The Italian, Palestinian, and Israeli isolates increased to 2, 3 and 33, respectively. The Palestinian and Israeli isolates were mainly isolated in the middle of the country (Additional file 2). The mutation (22295C > A) created a new potential N-glycosylation site at position 245 that changed the amino acid Histidine (H) in the reference strain to Asparagine (N) and this might influence the quality of antibody recognition in COVID-19 virus. The ten Palestinian strains contained 129 mutations with an average of 12.9 mutations (Additional file 3).

### Phylogenetic analysis and haplotype networking

**Fig. 1:**
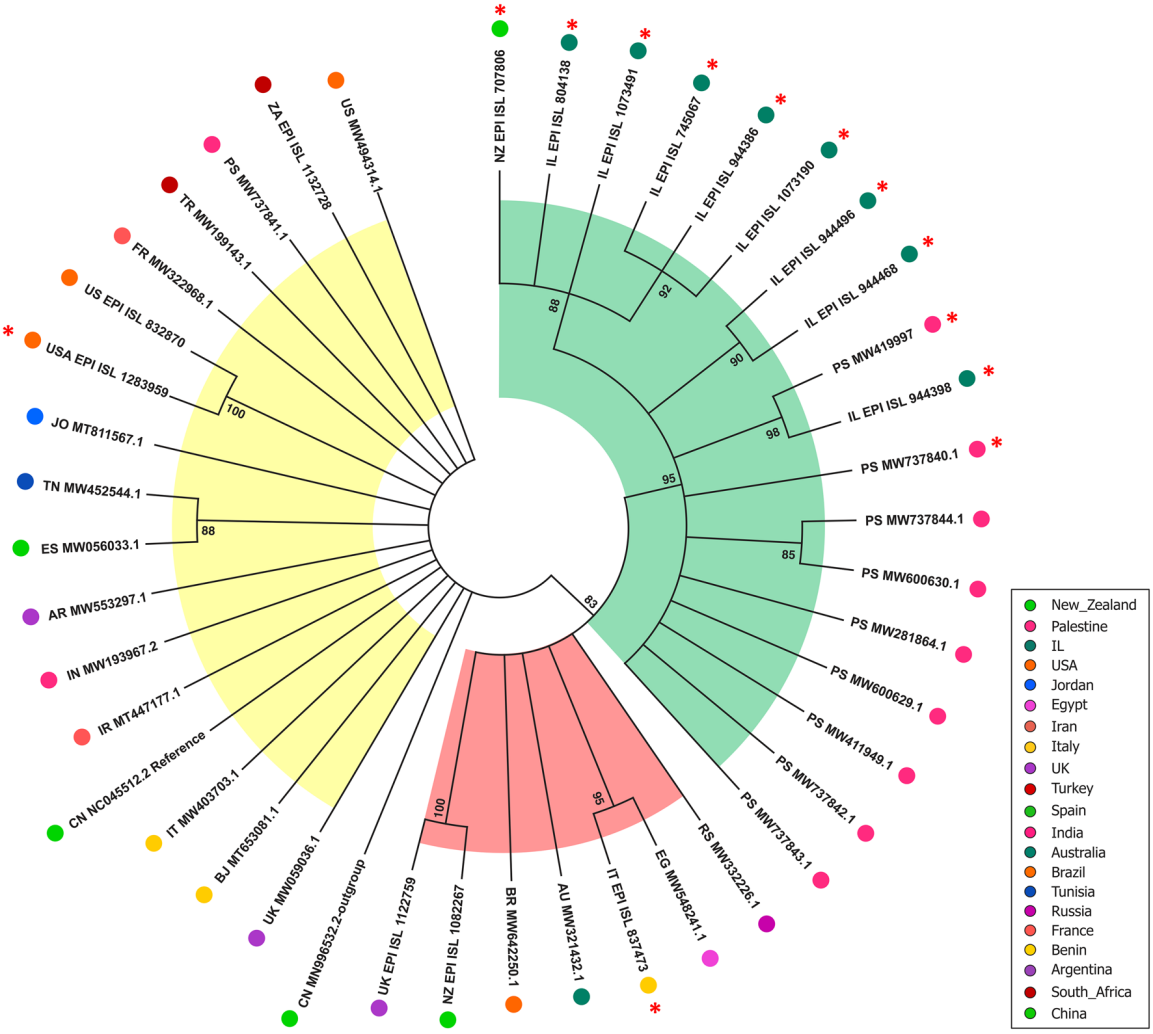
Circular consensus Maximum-likelihood phylogenetic tree (1000 replicates) based on SARS-CoV-2 whole genome

Maximum likelihood (ML) phylogenetic tree of complete genome sequences was included in the tree construction. The tree showed 3 clusters (Fig. 1). Cluster-III (green) mainly consisted of the nine Palestinian and eight Israeli isolates following a geographical distribution pattern, in addition to a bizarre sequence that was found in a News Zealander sample. Eleven out of 18 genome sequences (61%) in cluster-III contained the unique Middle Eastern spike mutation H245N compared to one (14%) in cluster-II (red) and one (6%) in cluster-I (yellow). Clusters-I and II did not follow any geographical distribution patterns.

**Fig. 2:**
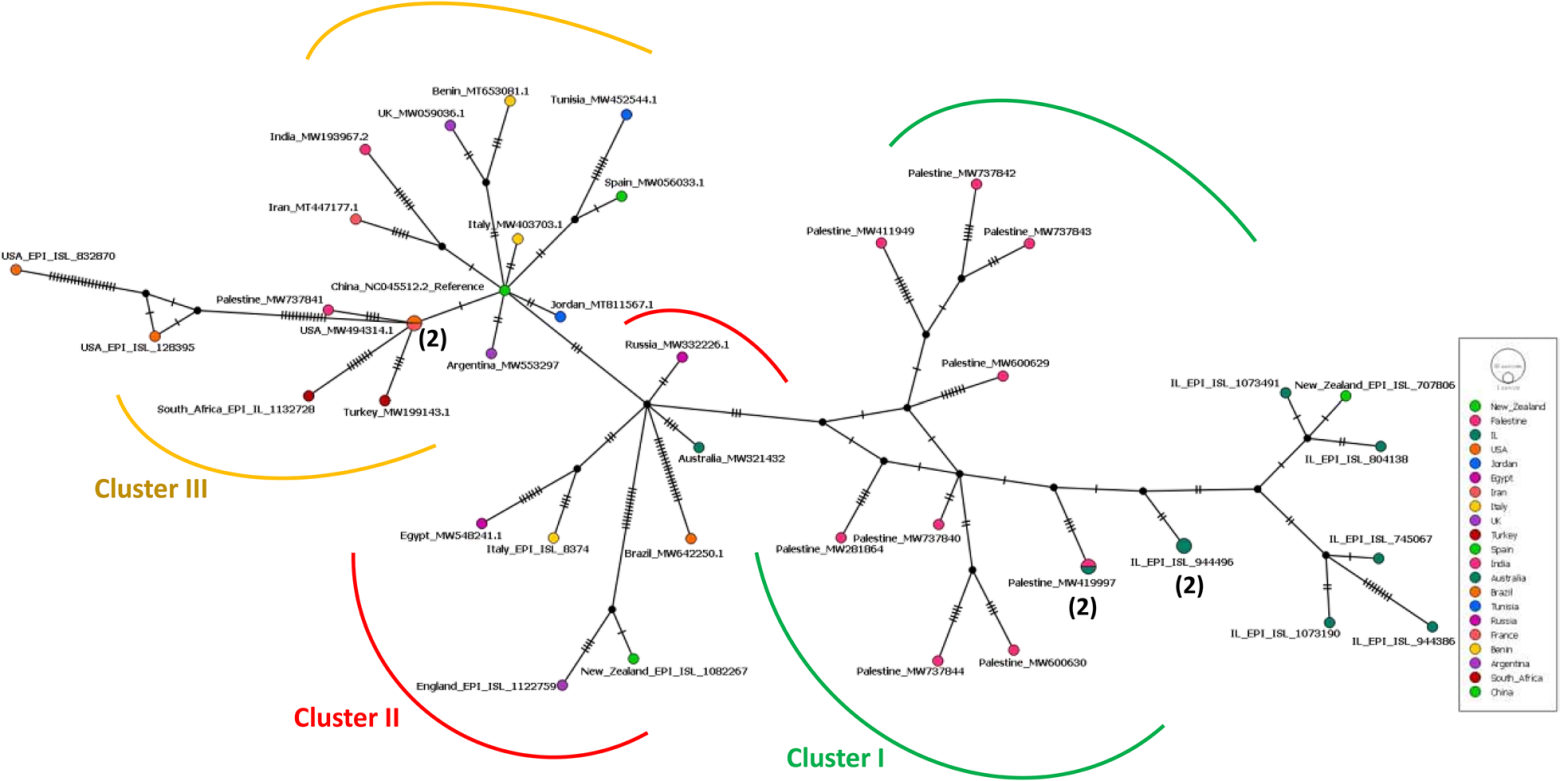
Median-joining haplotype network of SARS-CoV2 haplotypes constructed using PopArt 1.7

The median-joining haplotype network (MJN) constructed by PopArt 1.7 using the same 42 taxa used in constructing the MJ tree produced a total of 39 active haplotypes. The haplotype network analysis supported the phylogenetic tree in showing three clusters. In cluster-III, a star pattern was formed around the Wuhan reference strain (hCoV-19/Wuhan/Hu-1/2019) which extended to cluster-II and cluster-I consequently (Fig. 2).

The peripheral haplotypes were shown to be more than a single nucleotide variation (SNV) away from the central haplotypes such as Wuhan reference haplotype, the French-American haplotype, and the hypothetical haplotypes (black circles) (Fig. 2). Cluster-I contained more hypothetical haplotypes than cluster-II and III.

### Population genetics of SARS-CoV-2

Population diversity indices and neutrality tests were carried out for the complete SARS-CoV-2 genomes based on the three clusters produced by phylogenetic and haplotype network analyses. Cluster-I had the highest number of mutations (Eta) (120) compared to cluster-II and III. The average number of nucleotide differences between any two sequences (k) was 17.7 which is very high. The DnaSP ver. 6.12.03 estimated the total number of haplotypes for the three clusters at 39 with highest in cluster-I (h = 17) (Table 1). The three clusters had high haplotype diversity (Hd), but low nucleotide diversity (π). The total haplotype (gene) diversity (Hd) for the 42 viral genomes was 0.99 ± 0.00006. Concomitantly, the total nucleotide diversity per site (π) was 0.001 ± 0.0001, indicating low genetic diversity in the SARS-CoV-2 study genome bulk. Cluster-III (n = 17) with the Wuhan reference genome (hCoV-19/Wuhan/Hu-1/2019) as the radiating sequence contained the lowest haplotype-to-number (h:n) ratio of 0.88 compared to a ratio of 1 in the other two clusters. Tajima’s D and Fu-Li’s F tests showed statistically significant difference from neutral expectations, except cluster-II. Cluster- II did not depart significantly from neutrality (P > 0.01) (Table 1). The Fu-Li’s F values were in agreement of Tajima’s D.

**Table 1:** Genetic diversity and neutrality tests of the three probable clusters of the SARS-CoV-2 genomes included in the pilot survey

	Haplotype- nucleotide diversity							Neutrality tests	
Cluster	n	h	Eta	Hd ± SD	π ± SD	K	S	Tajima’s D	Fu-Li’s F
Cluster-I	17	17	120	1.00 ± 0.002	0.0007 ± 0.0	17.96	118	− 2.11*	− 2.74*
Cluster-II	7	7	91	1.00 ± 0.009	0.001 ± 0.00	30.09	91	− 1.11	− 1.17
Cluster-III	18	16	83	0.98 ± 0.000	0.0007 ± 0.0	13.56	83	− 1.83*	− 2.66**
Total	42	39	217	0.99 ± 0.000	0.001 ± 0.00	17.61	214	− 2.40**	− 4.28**

n: Number of sequences, h: Number of Haplotypes, Hd: Haplotype (gene) diversity, π: Nucleotide diversity (per site) [17], K: Average number of nucleotide differences between two randomly chosen sequences within the population [18], S: Number of variable/segregating sites. Eta: Total number of mutations. **P < 0.01, *P < 0.05

Inter-population pairwise genetic distance (Fst) among the three SARS-CoV-2 clusters ranged between 0.16 and 0.43 with a migration rate (Nm) estimating between 0.72 and 1.63 (Additional file 3). The pairwise genetic distance (Fst) was largest between cluster- I and III with a value of 0.41 (> 0.25) and lowest between cluster- I and II (0.16). The migration rate (Nm) estimates were inversely proportional to Fst values in which when genetic differentiation (Fst) was maximum, the migration rate (Nm) among subpopulations was minimum and vice versa. However, other inter-population genetics indices including Kxy, Dxy, Da, Gst followed a low trend indicating a structure with low genetic differentiation among the three probable clusters I, II and III (Additional file 4).

### Discussion

The study reports the complete genome sequence of SARS-CoV-2 from ten Palestinian SARS-CoV-2 isolated from COVID-19 patients in Palestine. The maximum likelihood phylogenetic tree and MJ haplotype network congruently showed that the nine out of ten (90%) Palestinian SARS-CoV-2 genome sequences congregate in one cluster together with Israeli genome sequences (Fig. 1). The Palestinian and Israeli isolates have spread over the same geographical domains because both variants were extremely contagious as one Palestinian and one Israeli isolates sharing the same haplotype node (Fig. 2). However, among the three clusters discovered in the phylogenetic analysis, cluster-I had the highest genetic variation evinced by indices of diversity, increased hypothetical nodes in the haplotype network, and significantly negative Tajima’s D and Fu-Li’s F values (Additional file 4, Fig. 2). This may reflect the different origins of SARS-CoV-2 or high migration rate (gene flow) within the cluster itself. Although cluster-III originated from different geographical areas, it was less genetically diverse than cluster-I as it had the lowest haplotype-to-number ratio (h:n = 0.88) along with minimum number of mutations (83). Nevertheless, and despite having lower genetic variation, cluster-III is still considered a well-established cluster. This was evidenced by the high pairwise genetic distance (Fst) between clusters I and III with a value of 0.41 (> 0.25), low gene flow between the two clusters (Nm = 0.72), and significant departure from neutrality as shown by negative Tajima’s D and Fu-Li’s F values (Table 1, Additional file 4). The low gene flow between cluster-I (Palestinian-Israeli) and cluster III that contains the Wuhan reference strain as the center of cluster indicates low host movement.

Cluster-I had higher genetic proximity to cluster-I rather than cluster-III due to low Fst value (0.16) high Nm value (1.63), and insignificant Tajima’s D and Fu-Li’s F values indicating relatively high gene flow between the two clusters. The high gene flow (gene migration, Nm), movement of genes from one population to another geographically distant population, between clusters I and II indicates host to host movement of host facilitated by international travel or commerce or can be due to geographical dispersion between clusters. The natural selection and selective sweep are less probable causes of low genetic diversity in cluster-II due to recent emergence of the SARS-CoV-2 pandemic. The most plausible explanation of the observed genetic variation within clusters I and III is the recent expansion of the SARS-COV-2 population all over the globe after a certain bottleneck event such as the possible recent transmission of virus from horseshoe bat (*Rhinolophus *spp.) to human [19]. The relatively high haplotype diversity (Hd) with simultaneously low nucleotide diversity (π) is another indicator of the recent population expansion of SARS-CoV-2 that explains low genetic variation (Table 1).

The GISAID database showed that the spike mutation H245N first reported in a Palestinian patient early November 2020 was spread in five countries (PS, IT, IL, NZ, and US) (Additional file 2). The mutation resulted in amino acid change in the conserved spike glycoprotein which plays a pivotal role in the viral mimicry used by SARS-CoV-2 to evade host immune response, neutralize antibodies, increase viral transmissibility, and escalate vaccine escape [20, 21]. All ten study genome sequences contained the spike mutation D614G which is the most prevalent mutation (90.2%) reported in 132 countries which has been shown to increase infectivity of the virus by increasing cellular transduction [22]. However, none of the studied samples showed any of the prominent spike mutations such as the British (B.1.17), Brazilian (B.1.1.128) or South African (B. 1. 1. 351) [23, 24].

### Conclusion

This study reported the complete genome sequence, characterization, phylogenetic analysis, haplotype network analysis, and genetic diversity of a pilot group of 10 SARS-CoV-2 from Palestine. A spike mutation H245N was first reported in one of the Palestinian isolates. Palestinian and Israeli SARS-CoV-2 genome sequences clustered together. A more comprehensive study with larger sample size over an extended span of time is crucial to investigate the molecular epidemiology of SARS-CoV-2 in Palestine.

### Limitations

A limitation to our study is the small sample size of Palestinian SARS-CoV-2 genomes. This was evident by the highest number of hypothetical haplotypes shown in black circles in cluster-I of the haplotype network. The low number of genome sequences provided incomplete picture of the genetic relatedness between the genome sequences.

## Supplementary Information

**Additional file 1.** Spot mapping of the ten Palestinian COVID-19 samples by district. Map provided by the Applied Research Institute-Jerusalem (ARIJ: www.ARIJ.og).

**Additional file 2.** (A) Global geographical distribution of the spike mutation H245N (Map made with Khartis, a free online map source: https://www.sciencespo.fr/cartographie/khartis/en/) (B) Regional distribution of the same mutation in the Middle East. The mutation is restricted to Palestinian and Israeli patients. Red location icon indicates the first reported mutation, blue icons indicate the mutations thereafter, while the green icon indicates the most recent reporting of the mutation. The number in the location icon represents the number of mutations in the specific region.

**Additional file 3.** The distribution of mutations in the 10 Palestinian isolates along the SARS-CoV-2 genome schematic display. Mutation in red brackets indicate uniqueness, while bold and under lined is the Palestinian Spike mutation H245N.

**Additional file 4.** Population genetic differentiation and gene flow indices between the three SARS-CoV-2 probable clusters.

## Data Availability

All data generated or analyzed during the current study are available in (GISIAD, https://www.gisaid.org/) GISAID accession IDs: (EPI_ISL_402125, EPI_ISL_745067, EPI_ISL_804138, EPI_ISL_944468, EPI_ISL_944496, EPI_ISL_1073190, EPI_ISL_1073491, EPI_ISL_944398, EPI_ISL_944386, PI_ISL_837473, EPI_ISL_707806, EPI_ISL_752605). Data on NCBI: Genbank accession numbers: MW419997 (EPI_ISL_752605), MW737840 (EPI_ISL_1273092), MW737844 (EPI_ISL_1273096), MW600630 (EPI_ISL_1273099), MW281864 (EPI_ISL_649153), MW600629 (EPI_ISL_1273098), MW411949 (EPI_ISL_739661), MW737842 (EPI_ISL_1273094), MW737843 (EPI_ISL_1273095), and MW737841 (EPI_ISL_1273093).

## Abbreviations

SARS-CoV-2: Severe acute respiratory syndrome coronavirus 2
NGS: Next generation sequencing
COVID-19: Corona Virus Disease-2019
ML: Maximum likelihood
MJN: Median-joining haplotype network
