# Supplementary material for: Complete genome sequencing of SARS-CoV-2 strains: A pilot survey in Palestine reveals spike mutation H245N
Source: BMC Res Notes. 2021 Dec 23;14:466. doi: 10.1186/s13104-021-05874-4 (PMC8698662; doi:10.1186/s13104-021-05874-4)
Supplement: Supplementary file 1 — Additional file 1. Spot mapping of the ten Palestinian COVID-19 samples by district. Map provided by the Applied Research Institute-Jerusalem (ARIJ: www.ARIJ.og). [file 13104_2021_5874_MOESM1_ESM.docx]

| 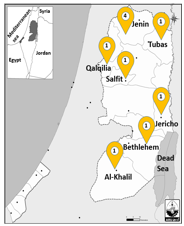 |
| --- |
| **Additional file. 1**. Spot mapping of the ten Palestinian COVID-19 samples by district. Map provided by the Applied Research Institute-Jerusalem (ARIJ: www.ARIJ.og). |
