# Supplementary material for: Complete genome sequencing of SARS-CoV-2 strains: A pilot survey in Palestine reveals spike mutation H245N
Source: BMC Res Notes. 2021 Dec 23;14:466. doi: 10.1186/s13104-021-05874-4 (PMC8698662; doi:10.1186/s13104-021-05874-4)
Supplement: Supplementary file 2 — Additional file 2. (A) Global geographical distribution of the spike mutation H245N (Map made with Khartis, a free online map source: https://www.sciencespo.fr/cartographie/khartis/en/) (B) Regional distribution of the same mutation in the Middle East. The mutation is restricted to Palestinian and Israeli patients. Red location icon indicates the first reported mutation, blue icons indicate the mutations thereafter, while the green icon indicates the most recent reporting of the mutation. The number in the location icon represents the number of mutations in the specific region. [file 13104_2021_5874_MOESM2_ESM.docx]

| 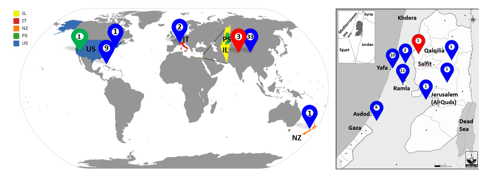 | |
| --- | --- |
| **(A)** | **(B)** |
| **Additional file 2**. (A) Global geographical distribution of the spike mutation H245N (Map made with Khartis, a free online map source: https://www.sciencespo.fr/cartographie/khartis/en/) (B) Regional distribution of the same mutation in the Middle East. The mutation is restricted to Palestinian and Israeli patients. Red location icon indicates the first reported mutation, blue icons indicate the mutations thereafter, while the green icon indicates the most recent reporting of the mutation. The number in the location icon represents the number of mutations in the specific region. | |
|  | |
