# Supplementary material for: Complete genome sequencing of SARS-CoV-2 strains: A pilot survey in Palestine reveals spike mutation H245N
Source: BMC Res Notes. 2021 Dec 23;14:466. doi: 10.1186/s13104-021-05874-4 (PMC8698662; doi:10.1186/s13104-021-05874-4)
Supplement: Supplementary file 3 — Additional file 3. The distribution of mutations in the 10 Palestinian isolates along the SARS-CoV-2 genome schematic display. Mutation in red brackets indicate uniqueness, while bold and under lined is the Palestinian Spike mutation H245N. [file 13104_2021_5874_MOESM3_ESM.docx]

| 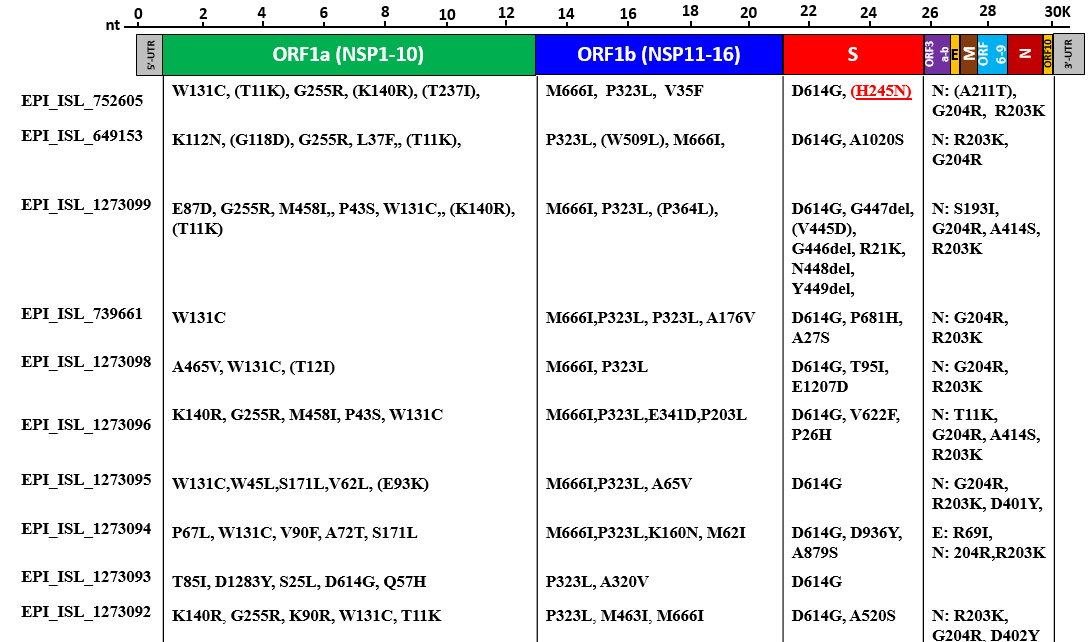 |
| --- |
| **Additional file 3**. The distribution of mutations in the 10 Palestinian isolates along the SARS-CoV-2 genome schematic display. Mutation in red brackets indicate uniqueness, while bold and under lined is the Palestinian Spike mutation H245N |
