# Supplementary material for: Complete genome sequencing of SARS-CoV-2 strains: A pilot survey in Palestine reveals spike mutation H245N
Source: BMC Res Notes. 2021 Dec 23;14:466. doi: 10.1186/s13104-021-05874-4 (PMC8698662; doi:10.1186/s13104-021-05874-4)
Supplement: Supplementary file 4 — Additional file 4. Population genetic differentiation and gene flow indices between the three SARS-CoV-2 probable clusters. [file 13104_2021_5874_MOESM4_ESM.docx]

**Additional file 4**: Population genetic differentiation and gene flow indices between the three SARS-CoV-2 probable clusters

| Pop 1 | Pop 2 | Fst | Nm | Kxy | Dxy | Gst | Da |
| --- | --- | --- | --- | --- | --- | --- | --- |
| Cluster-I | Cluster-II | 0.16 | 1.63 | 18.45 | 0.001 | 0.012 | 0.0002 |
| Cluster-I | Cluster-III | 0.41 | 0.72 | 20.55 | 0.001 | 0.005 | 0.0005 |
| Cluster-II | Cluster-III | 0.28 | 1.29 | 20.94 | 0.001 | 0.006 | 0.0003 |

Fst: Wright’s F-statistics, pairwise genetic distance, Nm: Gene flow and population migration among populations, Nm=(1-Fst)/2Fst haploid, Nm=(1-Fst)/4Fst diploid, Kxy: The average number of nucleotide differences between populations 1 and 2. Dxy: The average number of nucleotide substitutions per site between populations 1 and 2, Da: The number of net nucleotide substitutions per site between populations 1 and 2, Gst: Genetic differentiation index based on the frequency of haplotypes.
